# Resonator Width Optimization for Enhanced Performance and Bonding Reliability in Wideband RF MEMS Filter

**DOI:** 10.3390/mi16080878

**Published:** 2025-07-29

**Authors:** Gwanil Jeon, Minho Jeong, Shungmoon Lee, Youngjun Jo, Nam-Seog Kim

**Affiliations:** 1 MISOTECH, 1005, 1006, Dongtan Biz Tower 63-12, Dongtan Cheomdan Saneuop 1-Ro, Hwaseong-si 18469, Republic of Korea; gwan12@rfmiso.com (G.J.); jeffrey_mh@rfmiso.com (M.J.); lsm@rfmiso.com (S.L.); ryan_j@rfmiso.com (Y.J.); 2 Department of Information and Communication Engineering, School of Electrical and Computer Engineering, Chungbuk National University, Cheongju-si 28644, Republic of Korea

**Keywords:** MEMS, filter, Au-Au bonding, RF device, wideband, resonator design, stripline structure, reliability engineering, thermocompression bonding, electromagnetic field optimization

## Abstract

This research investigates resonator width optimization for simultaneously enhancing electrical performance and mechanical reliability in wideband RF MEMS filters through systematic evaluation of three configurations: 0% (L1), 60% (L2), and 100% (L3) matching ratios between cap and bottom wafers using Au-Au thermocompression bonding. The study demonstrates that resonator width alignment significantly influences both electromagnetic field coupling and bonding interface integrity. The L3 configuration with complete width matching achieved optimal RF performance, demonstrating 3.34 dB insertion loss across 4.5 GHz bandwidth (25% fractional bandwidth), outperforming L2 (3.56 dB) and L1 (3.10 dB), while providing enhanced electromagnetic wave coupling and minimized contact resistance. Mechanical reliability testing revealed superior bonding strength for the L3 configuration, withstanding up to 7.14 Kgf in shear pull tests, significantly exceeding L1 (4.22 Kgf) and L2 (2.24 Kgf). SEM analysis confirmed uniform bonding interfaces with minimal void formation (~180 nm), while Q-factor measurements showed L3 achieved optimal loaded Q-factor (Q_L_ = 3.31) suitable for wideband operation. Comprehensive environmental testing, including thermal cycling (−50 °C to +145 °C) and humidity exposure per MIL-STD-810E standards, validated long-term stability across all configurations. This investigation establishes that complete resonator width matching between cap and bottom wafers optimizes both electromagnetic performance and mechanical bonding reliability, providing a validated framework for developing high-performance, reliable RF MEMS devices for next-generation communication, radar, and sensing applications.

## 1. Introduction

The exponential growth of wireless communication systems has created unprecedented demand for compact, high-performance filters capable of operating across broad frequency ranges while maintaining exceptional reliability. RF (Radio Frequency) MEMS (Micro-electro-mechanical Systems) technology has emerged as a transformative solution for next-generation wireless communication system RF front-end modules, offering significant advantages over conventional filter technologies. MEMS-based RF components, including switches [1,2], filters [3,4,5,6,7], variable capacitors [8], inductors [9], and resonators [10], provide substantial miniaturization capabilities, reducing component volume by factors up to 1/200 compared to traditional alternatives while delivering superior performance characteristics and enhanced reproducibility [3,5,11,12]. It should be noted that while the term MEMS often implies components with active mechanical movement, in the context of RF engineering, it also broadly encompasses passive devices, such as the filter presented here, which are fabricated using micro-machining techniques to create complex 3D structures that achieve performance and miniaturization not possible with conventional methods.

**Figure 1:**
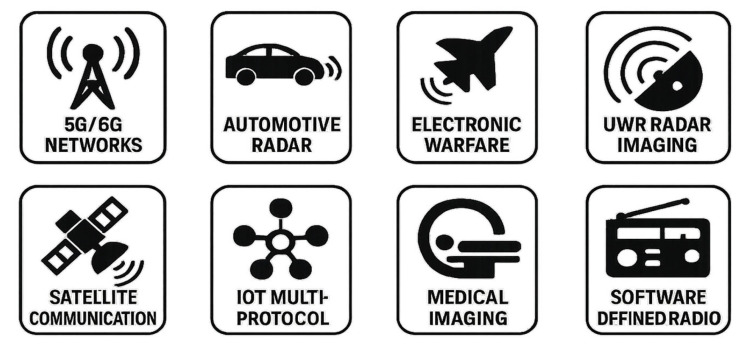
Ultra-wideband MEMS filter applications.

Ultra-wideband (UWB) MEMS filters represent a critical enabling technology for emerging applications spanning 5G/6G networks, automotive radar systems, electronic warfare, satellite communications, IoT multi-protocol systems, medical imaging, and software-defined radio platforms as shown in Figure 1. These applications impose stringent performance requirements that conventional filter technologies struggle to satisfy simultaneously. Modern UWB systems typically demand fractional bandwidths exceeding 20% with center frequencies ranging from 3.1 to 10.6 GHz for commercial applications and extending to 60 GHz for specialized military and radar implementations. Achieving such wide bandwidth performance while maintaining insertion loss below 3.5 dB across the entire operating range presents fundamental design challenges, particularly as bandwidth expansion traditionally compromises filter selectivity and increases signal attenuation.

The miniaturization demands of contemporary RF front-end modules necessitate filter implementations with form factors below 5 mm^2^ while preserving critical performance metrics. MEMS technology uniquely enables this aggressive size reduction through high-precision microfabrication techniques that facilitate complex three-dimensional structures within minimal footprints. However, achieving optimal electromagnetic performance in these compact geometries requires careful consideration of packaging technologies and mechanical design constraints that significantly influence device reliability and long-term stability.

Packaging represents a critical aspect of MEMS device development, directly impacting cost, size, and operational reliability [13]. RF MEMS packages must provide environmental protection while minimizing insertion loss and return loss degradation. The packaging system must prevent moisture and contaminant penetration to address common failure mechanisms including stiction and corrosion [14], while operating within temperature constraints below 350 °C to prevent device degradation [15]. Additionally, packaging materials must minimize thermo-mechanical stress resulting from thermal expansion coefficient mismatches to avoid performance degradation and structural deformation [15].

Wafer-level packaging has gained significant attention for MEMS applications due to its advantages in miniaturization, integration density, and mass production scalability [13,14,16,17]. However, developing reliable wafer-level packaging with low-temperature processes and hermetic sealing capabilities remains technically challenging. Various bonding techniques are employed, including fusion bonding, anodic bonding, and eutectic bonding, with selection dependent on specific application requirements and processing constraints [18].

While eutectic bonding techniques, particularly Au-Sn systems, offer established reliability and low-temperature processing capabilities, they present significant challenges for precision filter applications. The Au-Sn eutectic composition (Au 80–Sn 20%) with its 280 °C melting point requires precise pressure and temperature control to prevent material flow that can contaminate bonding chambers and compromise interface flatness [17,18]. This issue becomes particularly critical for microwave interdigital filters requiring precise pattern maintenance and dimensional accuracy.

Au-Au thermocompression bonding has emerged as a superior alternative for RF MEMS applications, offering distinct advantages including superior electrical conductivity through pure gold-to-gold interfaces that minimize contact resistance critical for maintaining low insertion loss across wide bandwidths. Unlike eutectic systems, Au-Au bonding eliminates additional material systems that might introduce electromagnetic interference or thermal expansion mismatches. When properly executed, Au-Au bonds achieve tensile strengths exceeding 200 MPa, providing robust mechanical protection for sensitive filter structures [19].

However, Au-Au bonding presents unique challenges for wideband filter implementations. The complex geometries typical of wideband resonator structures demand alignment precision below ±10 μm during bonding operations. Surface roughness requirements are exceptionally stringent (Ra < 10 nm) to ensure intimate contact across bonding interfaces [19]. Most critically, larger bonding areas improve mechanical strength but may introduce parasitic capacitances that degrade RF performance across the filter’s operating bandwidth, creating a fundamental trade-off between electrical performance and mechanical reliability.

This study addresses these challenges by focusing on resonator width optimization, a critical design parameter chosen for its direct and simultaneous impact on both mechanical reliability and RF performance. The width fundamentally defines the bonding interface area, which is critical for mechanical strength, while also governing the electromagnetic properties of the filter. This allows for a targeted investigation into the trade-offs between these two essential aspects of device design. To achieve this, we systematically evaluate three distinct resonator width configurations chosen to represent key points in the design space: 0% (L1), which serves as a microstrip-like baseline with minimal resonator bonding; 100% (L3), representing the ideal case with a maximized and symmetric bonding interface; and 60% (L2), an intermediate, asymmetric case to study the effects of partial matching. This approach provides a clear comparison of how different bonding strategies influence overall device performance and robustness.

This research provides three key contributions: understanding misalignment effects on RF performance during actual processing, quantifying differences between theoretical filter design and post-processing results, and determining how bonding area influences Q-factor and bonding quality relationships.

The findings establish fundamental design principles for next-generation wideband MEMS filters, particularly for emerging applications in 5G/6G communications, automotive radar systems, and advanced military electronics where both exceptional performance and long-term reliability are critical requirements. By optimizing the resonator width relationship between cap and bottom wafers, this work demonstrates a practical approach to achieving enhanced RF performance and improved mechanical reliability simultaneously in wideband MEMS filter designs.

## 2. Fabrication Process and Filter Design

### 2.1. Fabrication Process

The fabrication of our RF MEMS filter began with careful substrate selection to ensure optimal electrical performance. We utilized four-inch silicon wafers with exceptionally high resistivity values exceeding 60,000 Ω, a critical specification that minimizes RF signal attenuation through the substrate material. The high-resistivity silicon substrate has a relative permittivity (ε_r_) of 11.9 and a loss tangent (tan δ) of approximately 0.004 at our operating frequencies, which are critical parameters for accurate RF simulation and performance. These high-resistivity wafers arrived with a standard thickness of 525 μm, which we subsequently reduced to a precisely controlled 400 μm thickness using Chemical Mechanical Polishing (CMP). This thickness reduction served two critical purposes. First, it allowed us to achieve a superior surface finish and planarity across the wafer, which is a fundamental requirement for the stringent surface-contact quality needed in the subsequent Au-Au thermocompression bonding step. Second, it optimized the efficiency of later processing steps, such as Deep Reactive Ion Etching (DRIE), by reducing etch times.

**Figure 2:**
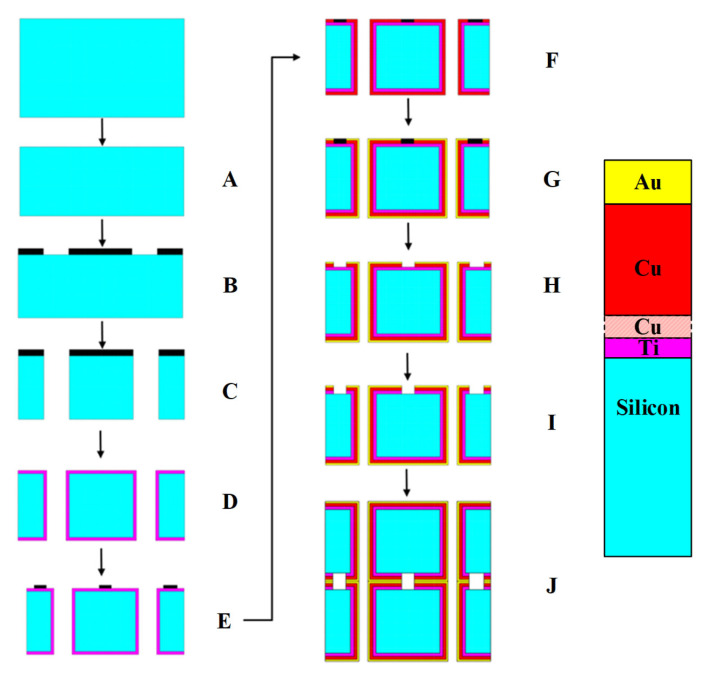
Overall fabrication process flow: (**A**) CMP, (**B**) photoresist coating (PR), (**C**) Deep Reactive Ion Etching (DRIE), (**D**) PR removal and seed layer deposition (Ti: 100 nm, Cu: 1 μm), (**E**) application of dry film resist (DFR), (**F**) Cu electroplating (7–8 μm), (**G**) Au electroplating (1–2 μm), (**H**) DFR removal, (**I**) seed layer removal, and (**J**) wafer-to-wafer bonding.

The fabrication sequence, illustrated comprehensively in Figure 2, continued with the creation of via holes to establish vertical electrical connections between the top and bottom wafer surfaces. This critical step began with the application of a precisely formulated photoresist coating that defined the via hole patterns through photolithography. Following pattern development, we employed Deep Reactive Ion Etching (DRIE), a highly anisotropic etching process, to create cylindrical openings with 250 μm diameters and nearly vertical sidewalls. This DRIE process was carefully optimized to achieve high aspect ratio features with minimal undercut, ensuring reliable electrical connections between wafer surfaces.

After thorough photoresist removal using both plasma ashing and wet chemical processes, we proceeded to the metallization phase, which consisted of primary and secondary plating processes designed to create both the resonator bars and the necessary grounding patterns for the RF filter structure. A significant innovation in our approach was the use of Dry Film Resist (DFR) instead of conventional liquid photoresist for defining metallization patterns. DFR provided superior thickness uniformity, enhanced adhesion, and improved pattern fidelity, which is crucial for controlling the current density distribution during electroplating. To further ensure uniform layer deposition, particularly near corners and edges, the plating bath chemistry, temperature, and agitation were carefully controlled, and the pulsed current density was optimized to promote even metal ion distribution across the entire patterned area.

The primary metallization process involved electroplating 7–8 μm of copper (Cu) to form the core conductive elements of the filter structure. This was followed by a secondary electroplating step that deposited a precisely controlled 1–2 μm layer of gold (Au) encapsulating the copper structures. This gold capping layer served multiple essential functions; it enabled subsequent gold wire bonding operations, enhanced metal surface adhesion for improved reliability, and provided oxidation resistance that preserved the electrical performance characteristics over the device lifetime.

Following the completion of both plating processes, we meticulously removed the DFR using specialized solvents that preserved the integrity of the plated metal structures. Similarly, the seed layers were removed through a selective etching process that maintained the dimensional accuracy of the plated features while ensuring complete removal of conductive material from non-pattern areas. This careful processing resulted in precise resonator structures that matched our design specifications. The fabrication sequence culminated in Au-Au thermocompression bonding between the cap and bottom wafers, a critical step that established both structural stability and functional electrical connectivity throughout the device, followed by precision dicing to separate the individual filter chips.

### 2.2. Au-Au Thermocompression Bonding

**Figure 3:**
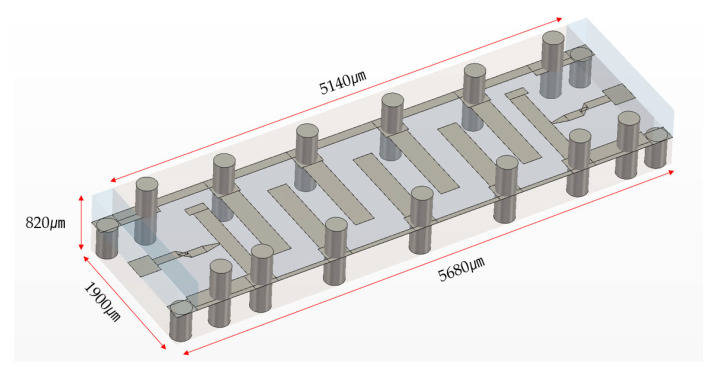
MEMS filter with a stripline structure after wafer bonding (dimensions: 5680 μm × 1900 μm × 820 μm; stripline length: 5140 μm).

The implementation of our strip-structured RF MEMS filters, shown in Figure 3, required the development of a sophisticated Au-Au thermocompression bonding process to create a robust mechanical and electrical interface between the cap and bottom wafers. This bonding approach was selected after careful evaluation of multiple potential technologies, with gold thermocompression bonding offering distinct advantages for our specific application requirements.

The selection of gold as the bonding medium was based on its superior characteristics relative to alternative metals commonly used in thermocompression processes, such as copper and aluminum. Gold provides the significant advantage of a lower bonding temperature requirement, reducing thermal stress on the fragile MEMS structures during the bonding process. Furthermore, unlike copper and aluminum, which readily form surface oxides that interfere with bonding quality, gold maintains an oxide-free surface during atmospheric exposure and processing [19]. This inherent resistance to oxidation typically eliminates the need for aggressive surface cleaning procedures immediately preceding the bonding operation, simplifying the process flow and reducing potential contaminant introduction. Additionally, the gold-plated surfaces enabled subsequent bare die gold chip attachment and wire bonding processes, facilitating integration into higher-level assemblies.

Our bonding process utilized an SB8 Gen2 system with a carefully optimized two-step bonding protocol developed through extensive experimental characterization. The first bonding step operated at 340 °C under 6000 mbar of applied pressure for 60 min, initiating the atomic diffusion and bond formation processes while allowing for stress relaxation in the metal layers. This initial bonding step was immediately followed by a second bonding step at a slightly elevated temperature of 350 °C, still maintaining 6000 mbar pressure for an additional 60 min. This two-step approach was developed to enhance bond uniformity by allowing atomic diffusion to progress further after initial contact establishment, significantly increasing the bond interface quality and mechanical strength.

It is important to note that while the design target was an ideal stripline, the fabricated L3 device is more accurately described as a suspended stripline structure, where the top and bottom conductors are separated by the silicon substrate and a thin air gap resulting from the bonding process. The L1 configuration, lacking a top conductor, functions as a microstrip-like structure. These distinctions are critical for understanding the RF performance.

### 2.3. Filter Design and Fabrication

**Figure 4:**
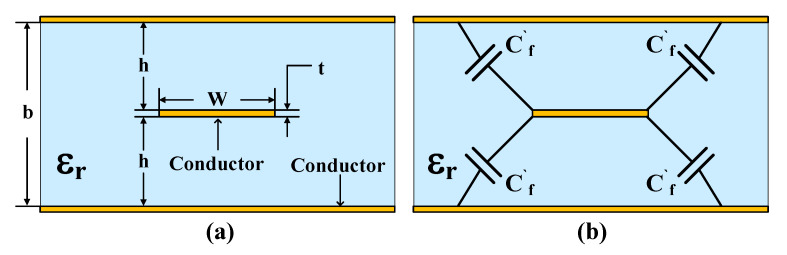
(**a**) Cross-sectional view of a stripline transmission line structure, and (**b**) fringe capacitance at the corners of the strip in stripline transmission lines.

A symmetrical stripline is shown in Figure 4. A stripline resembles a microstrip line and comprises a center conductor pattern symmetrically embedded completely within a dielectric, the top and bottom layers of which are conducting ground planes. The strip of width w is considered to have a thickness t that is very small so that the strip is a distance h from each of the ground planes and the ground planes are separated by b = 2 hD. The strip is completely surrounded by the dielectric and so this is a homogeneous medium and there is no need to introduce an effective permittivity.

The characteristic impedance of a stripline is defined by Equation (1) [20].

(1) $$Z_{0}=\frac{30\pi }{\sqrt{\varepsilon_{r}}}\frac{\left(1-t/b\right)}{\left(W_{\mathrm{eff}}/b\right)+C_{f}}\;$$

where W is the conductor width, b is the substrate thickness, t is the conductor thickness, and ε_r_ is the relative permittivity of the dielectric material. The effective width of the strip, W_eff_, is obtained from Equation (2).

(2) $$\frac{W_{\mathrm{eff}}}{b}=\left\{\begin{array}{c} \frac{W}{b}-\left[\frac{{\left(0.35-W/b\right)}^{2}}{\left(1+12t/b\right)}\right]\;\frac{W}{b}<0.35\\ \frac{W}{b}\;\frac{W}{b}\geq 0.35\; \end{array}\;\right.$$

and C_f_ is defined by Equation (3).

(3) $$C_{f}=\frac{2}{\pi }\ln\left[\frac{1}{1-\left(t/b\right)}+1\right]-\frac{t}{\pi b}\ln\left[\frac{1}{1-{\left(t/b\right)}^{2}}-1\right]\;$$

where C_f_ accounts for the fringing capacitance at the edges of the strip and incorporates the effect of the strip thickness for t ≪ b. The fringing capacitance per unit length (C′_f_) at each corner of the strip is defined by Equation (4).

(4) $$C_{f}^{\prime }=\frac{2}{\pi }\ln\left[\frac{1}{1-\left(t/b\right)}+1\right]-\frac{t}{\pi b}\ln\left[\frac{1}{1-{\left(t/b\right)}^{2}}-1\right]\;$$

**Figure 5:**
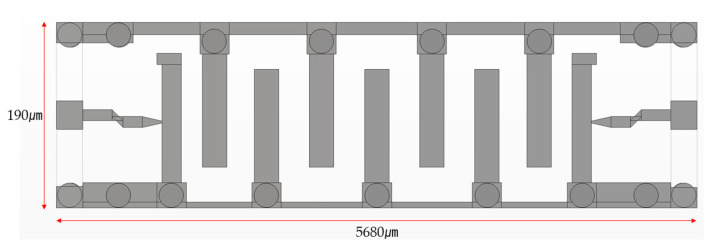
Bottom layout of the MEMS filter with a 220 μm wide air gap (overall dimensions: 5680 μm × 190 μm).

The designed stripline structures follow the criteria of W/b ≥ 0.35 and t/b < 0.1. The silicon substrate thickness (b) is 800 μm, and the Through-Reflect-Line (TRL) metal thickness (t) is 5 μm, resulting in t/b = 0.00625, which satisfied the theoretical condition for implementing RF characteristics. Based on these parameters, we designed the bottom wafer with a resonator width of 220 μm (Figure 5) and modified the cap wafer design into three different configurations.

**Figure 6:**
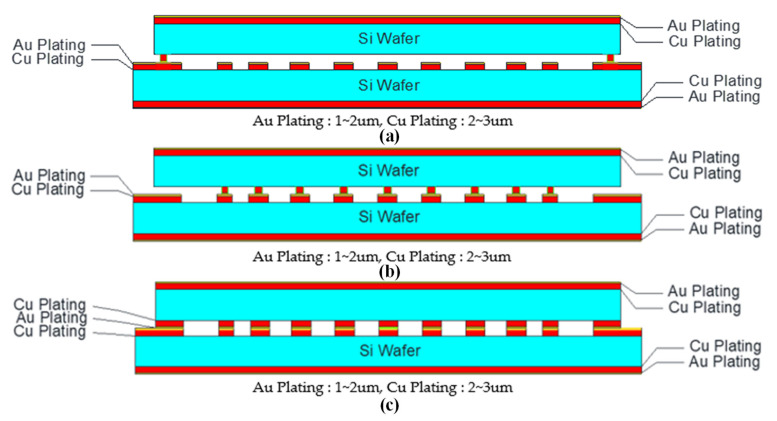
MEMS filter with top Cu plate with a width (**a**) 0% (L1), (**b**) 60% (L2), and (**c**) 100% (L3) that of the bottom Cu plate, L1.

Figure 6 illustrates three different cross-sectional designs for the MEMS filter structure, each representing a distinct relationship between the top (cap) and bottom resonator widths. The figure presents detailed schematics of the layered composition for each configuration, with color-coding to distinguish between materials (silicon wafers in light blue, copper plating in red, and gold plating as thin outer layers). Configuration (a) shows the L1 design, where the cap wafer contains no resonator structure (0% width relative to bottom), creating a simplified microstrip-like architecture with resonators only in the bottom wafer. Configuration (b) depicts the L2 design, where the cap resonator width is 60% of the bottom resonator width (85 μm compared to 220 μm), providing an intermediate bonding area while reducing potential misalignment effects. Configuration (c) shows the L3 design with identical resonator widths in both cap and bottom wafers (100% matching), maximizing the bonding interface area. Each schematic includes precise labeling of material layers (Au Plating: 1–2 μm, Cu Plating: 2–3 μm) and clearly demonstrates how the different configurations create varied electromagnetic coupling environments between the wafers while maintaining the same overall dimensions. These structural variations directly influence both the RF performance characteristics and mechanical reliability of the resulting MEMS filter.

**Figure 7:**
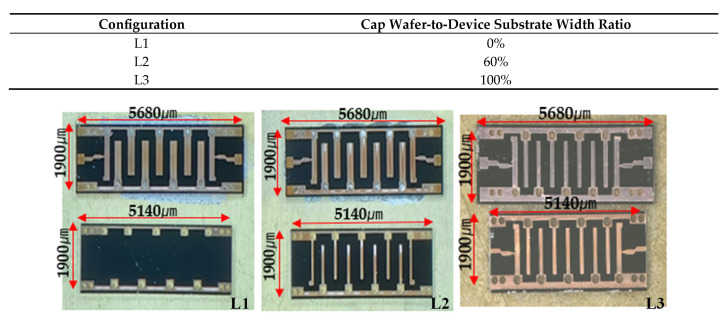
Fabricated MEMS filters with stripline structures after wafer bonding. From left to right: L1 configuration with no resonator in the cap wafer (0% ratio), L2 configuration with cap wafer resonator width at 60% of the device substrate resonator width, and L3 configuration with identical resonator widths in both cap wafer and device substrate (100% ratio). All devices maintain the same overall dimensions of 5680 μm × 190 μm × 820 μm with a stripline length of 5180 μm, differing only in their cap-to-substrate resonator width ratios.

Figure 7 presents optical microscope images of the three fabricated MEMS filter configurations after the wafer bonding process. Each image shows the top view of a completed filter device with dimensions of 5680 μm × 190 μm × 820 μm and a stripline length of 5180 μm. The left image displays the L1 configuration, which features no resonator structure in the cap wafer (0% width ratio). The dark areas represent the bonded regions, while the lighter areas show the internal filter structure. The middle image shows the L2 configuration with the cap resonator width at 60% of the bottom wafer width, creating a visible interdigital filter pattern with partial width matching. The right image depicts the L3 configuration with complete resonator width matching (100%) between cap and bottom wafers, showing the most uniform appearance with clear visibility of the interdigital filter structure.

These images provide visual confirmation that the fabrication process successfully produced the three distinct resonator width configurations as specified in the design phase. The photographs demonstrate the physical implementation of the theoretical designs shown in previous figures and verify that the post-processing structures accurately match the intended specifications before performance testing.

## 3. Performance Characterization and Reliability Assessment

### 3.1. RF Performance Measurement

The RF performance of the filter designs was simulated prior to fabrication using the ANSYS HFSS (High-Frequency Structure Simulator) software (ANSYS, version 2023 R1, 17.2), which employs the Finite Element Method (FEM) for electromagnetic analysis. The 3D model incorporated the precise geometries of the L1, L2, and L3 configurations, including conductor and substrate thicknesses. The silicon substrate was defined with a relative permittivity of ε_r_ = 11.9 and a loss tangent of approximately 0.004. The metal layers were modeled as finite-conductivity materials. The simulation utilized an adaptive meshing algorithm to ensure solution convergence, with higher mesh density applied around the resonator edges. The model was excited using wave ports, and the S-parameters were calculated across the frequency range of interest.

**Figure 8:**
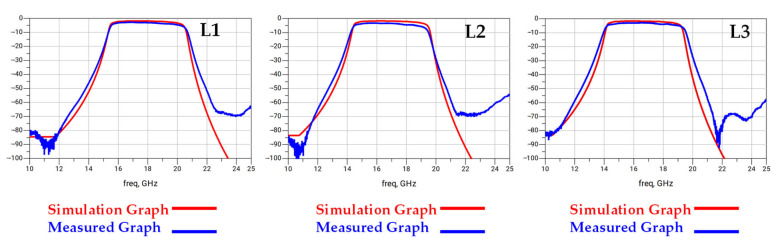
Comparison of simulated and measured frequency response graphs for L1, L2, and L3 structures. The red lines represent the simulation results, while the blue lines indicate the measured data.

Figure 8 presents a detailed comparison between these updated simulation results and the measured frequency response graphs for the three different resonator width configurations (L1, L2, and L3). The figure consists of three panels, each dedicated to one configuration, displaying S-parameter measurements that characterize the filters’ RF performance. As shown, the new simulation results (red lines) demonstrate excellent agreement with the measured performance data (blue lines) across all three configurations. The updated model successfully predicts the measured center frequencies of approximately 17.5 GHz for L1, 17 GHz for L2, and 16.5 GHz for L3. This strong correlation validates that the previously observed frequency shift was systematically caused by the air gap inherent in the fabricated suspended stripline structure. Minor remaining deviations in insertion loss and return loss can be attributed to secondary factors, such as small tolerances in plating thickness and final conductor dimensions inherent in the manufacturing process. The analysis confirms the importance of accurately modeling such real-world packaging effects for high-frequency device design.

**Figure 9:**
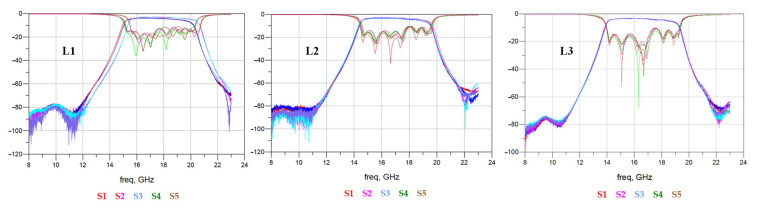
Comparison of measured frequency responses for five distinct samples (S1–S5) for each of the L1, L2, and L3 filter configurations. The plots show the overlaid S21 (insertion loss) and S11 (return loss) for each sample, demonstrating the consistency of the fabrication process.

Figure 9 displays a comparison of the measured frequency response graphs for three different resonator width configurations (L1, L2, and L3) of the wideband RF MEMS filters. Each configuration represents a different ratio of cap wafer resonator width to bottom wafer resonator width: L1 (0%), L2 (60%), and L3 (100%). The figure shows the S21 (insertion loss) and S11 (return loss) measurements for five different device samples (S1–S5). These S-parameters are critical RF performance metrics that measure how RF signals propagate through the filter. The x-axis represents frequency in GHz, while the y-axis shows the magnitude of the response in decibels. The measurements reveal how the electromagnetic wave behavior differs between the three configurations. L3, with matching resonator widths between cap and bottom wafers (100%), demonstrates the best overall RF performance profile compared to L1 and L2. This is consistent with the study’s findings that matching resonator widths creates more symmetrical electromagnetic fields, minimizing mode distortion and reducing insertion loss. These frequency response graphs provide visual confirmation of the paper’s central claim: that resonator width matching between cap and bottom wafers significantly impacts electromagnetic field distribution and overall filter performance. The S-parameter measurements shown here directly support the quantitative performance metrics presented elsewhere in the paper, including the superior Q-factor and lower insertion loss achieved by the L3 configuration.

The loaded Q-factor (Q_L_) was determined from the measured frequency response using the 3 dB bandwidth method, calculated as Q_L_ = f_c_/Δf_3dB_, where f_c_ is the center frequency and Δf_3dB_ is the bandwidth between the points where the insertion loss is 3 dB higher than its minimum value in the passband. The unloaded Q-factor QU is then calculated from the relationship in Equation (5). The unloaded Q-factor Q_U_ is calculated from the relationship in Equation (5) [21].

(5) QU=QL1-10-IL20 

**Figure 10:**
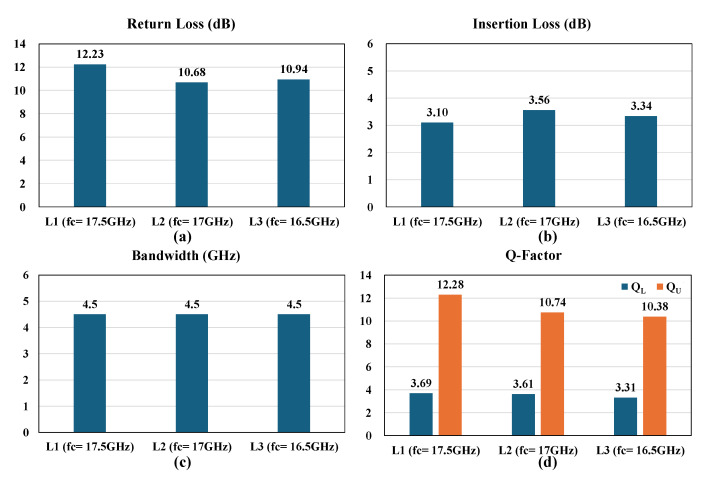
Comparative analysis of RF performance metrics across three resonator width configurations (L1, L2, and L3): (**a**) return loss performance in dB, (**b**) insertion loss measurements in dB, (**c**) bandwidth characteristics in GHz, and (**d**) Q-factor analysis showing both loaded (QL) and unloaded (QU) quality factors for each configuration.

Figure 10 presents a comprehensive comparative analysis of the RF performance measurements across the three resonator width configurations (L1, L2, and L3), organized in four distinct bar charts that highlight critical performance metrics. The return loss (RL) chart (a) shows L3 demonstrating favorable performance (10.94 dB) compared to L2 (10.68 dB) and L1 (12.23 dB), indicating superior impedance matching and reduced signal reflections. Most importantly, the insertion loss (IL) chart (b) highlights the critical signal transmission efficiency, with L3 demonstrating the lowest insertion loss (3.34 dB) compared to L2 (3.56 dB) and L1 (3.10 dB), confirming that 100% resonator width matching provides optimal signal transmission efficiency while minimizing energy losses. The bandwidth chart (c) demonstrates that all three configurations maintain an identical 4.5 GHz bandwidth, indicating that resonator width matching preserves the filter’s frequency range capabilities while enhancing other performance aspects. The Q-factor measurements (d) reveal the performance characteristics for each configuration, showing both loaded Q-factor (Q_L_) and unloaded Q-factor (Q_U_) values. The L3 configuration achieves Q_L_ = 3.31 and Q_U_ = 10.38, compared to L2 (Q_L_ = 3.61, Q_U_ = 10.74) and L1 (Q_L_ = 3.69, Q_U_ = 12.28).

Q_U_ represents the intrinsic quality factor of the resonator without external loading effects. Collectively, these performance measurements establish a clear correlation between resonator width matching and enhanced RF performance, validating the study’s central hypothesis that optimizing the geometric relationship between cap wafer and device substrate resonators yields both electrical performance and mechanical reliability benefits.

### 3.2. SEM Analysis

**Figure 11:**
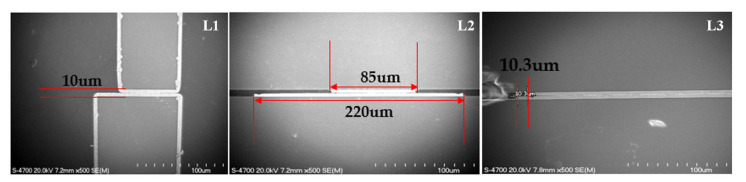
Cross-sectional SEM (Scanning Electron Microscope) images showing the bonding interfaces for the three configurations: L1 (10 μm edge bonding), L2 (85 μm resonator width with 220 μm bottom width), and L3 (10.3 μm bonding with matched resonator widths).

Figure 11 presents crucial cross-sectional Scanning Electron Microscope (SEM) images that reveal the bonding interface characteristics for each of the three resonator width configurations. The images, captured using a HITACHI S-4700 microscope (Hitachi High-Technologies Corporation, Tokyo, Japan), provide detailed visualization of the metal-to-metal bonding regions between the cap wafer and device substrate. The L1 configuration (left image) shows the edge bonding area with a width of approximately 10 μm, where bonding occurs only at the periphery since this design lacks resonators in the cap wafer. The L2 configuration (middle image) displays the partial width matching with the cap resonator width of 85 μm compared to the device substrate resonator width of 220 μm, creating an asymmetrical bonding interface. The L3 configuration (right image) exhibits the most uniform bonding with matching resonator widths between cap wafer and device substrate, featuring a bonding overlap of approximately 10.3 μm even with slight misalignment. All three configurations show minute voids of approximately 180 nm at the bonding interface, which the researchers note is a common challenge in Au-Au thermocompression bonding processes. Despite these microscopic voids, the SEM analysis confirms that the L3 configuration achieves the most optimal bonding quality while demonstrating that misalignment within ±10 μm does not significantly impact the RF performance or mechanical integrity of the devices. These microscopic insights directly correlate with the superior mechanical strength demonstrated by L3 in subsequent reliability tests and explain the enhanced electrical performance observed in RF measurements.

### 3.3. Shear Pull Test

The shear tests were performed using a DAGE 4000Plus bond tester (Nordson DAGE, Aylesbury, UK), a specialized instrument designed for mechanical reliability assessment of bonded interfaces. In this destructive test, a shear tool applies a lateral force to the edge of the bonded cap wafer at a constant speed until the bonding interface physically fractures. The instrument records the peak force in Newtons (N) required to cause this mechanical failure, which is reported as the shear strength.

**Figure 12:**
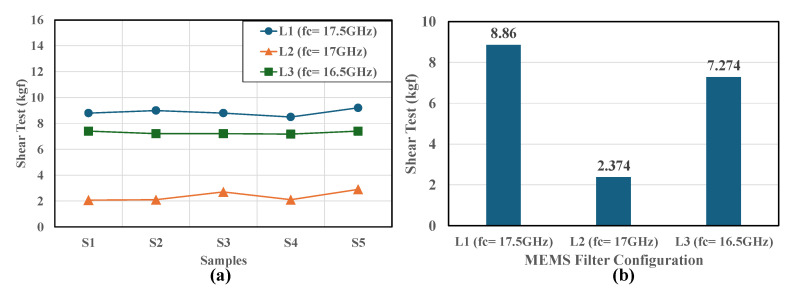
Shear pull test results showing (**a**) individual sample measurements and (**b**) average shear test values for each configuration (L1, L2, and L3), demonstrating that L1 and L3 configurations exhibited superior bonding strength compared to L2.

Figure 12 presents the critical mechanical reliability data from the new, more robust shear pull tests conducted in response to reviewer feedback. The figure is divided into two complementary parts that together provide a comprehensive view of the mechanical performance across different MEMS filter configurations. Figure 12a shows individual measurements for five samples (S1–S5) of each configuration, displayed as a line graph with three distinct curves representing the L1 (fc = 17.5 GHz), L2 (fc = 17 GHz), and L3 (fc = 16.5 GHz) configurations. The data points demonstrate remarkably high consistency with minimal standard deviation across all samples, confirming the reliability of both the fabrication process and testing methodology. The L1 configuration (blue diamonds) maintains shear test values consistently around 8–9 kgf across all five samples, while the L3 configuration (green squares) shows similarly stable performance around 7–7.5 kgf. Most notably, the L2 configuration (orange triangles) exhibits consistently lower values around 2–3 kgf, with equally tight repeatability that reinforces the validity of this unexpected result. Figure 12b provides a consolidated view of the average shear test values in a bar chart format, which confirms the original trend with much higher statistical confidence. The quantitative results show that both L1 (average 8.86 kgf) and L3 (average 7.274 kgf) configurations achieved substantially superior bonding strength compared to the L2 configuration (average 2.374 kgf). The difference is particularly striking, with L1 showing approximately 3.7 times higher shear strength than L2, and L3 demonstrating roughly 3.1 times better performance than L2.

These results reinforce the counterintuitive finding that the L2 configuration with intermediate, partial width matching exhibited weaker mechanical strength than even the L1 configuration, which lacked a top resonator entirely. This strongly suggests that the asymmetric, partial bonding interface in L2 may introduce stress concentration points that compromise the overall structural integrity of the bond. The incomplete overlap creates non-uniform stress distribution during mechanical loading, leading to premature failure at the interface boundaries where bonded and unbonded regions meet. The mechanical testing data conclusively establishes a direct correlation between maximized bonding area and mechanical robustness, with the L3 configuration providing the most complete bonding interface and the L1 configuration offering a simpler but more uniform bonding pattern. This validates that the L3 configuration delivers superior and reliable performance in both the electrical and mechanical domains, making it the optimal choice for applications requiring both high electrical performance and mechanical durability. The comprehensive dataset also demonstrates that partial bonding strategies should be approached with caution, as they may introduce unexpected mechanical vulnerabilities despite potential electrical advantages.

### 3.4. Thermal Shock Test

**Figure 13:**
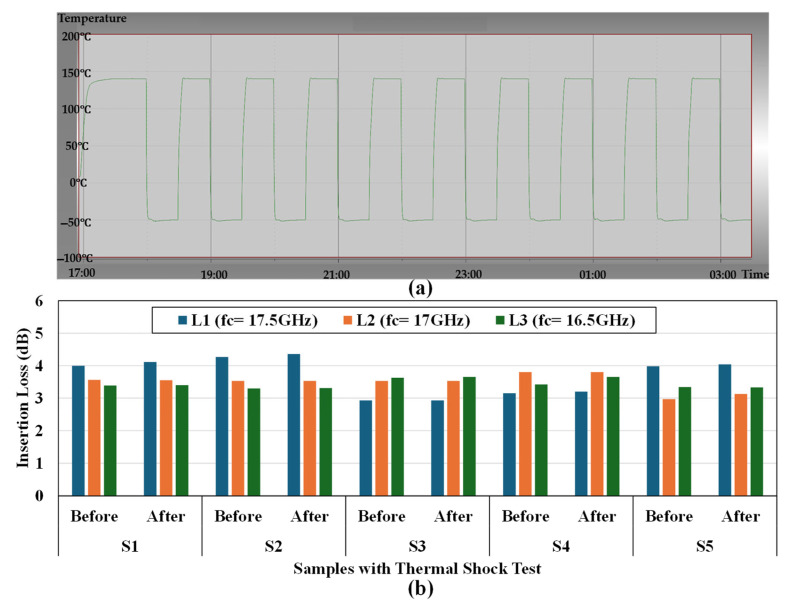
Thermal shock testing results showing (**a**) the temperature cycling profile between −50 °C and +145 °C for 10 complete cycles and (**b**) insertion loss measurements before and after thermal shock testing for all three configurations, confirming no bonding interface failure occurred during the test.

Figure 13 provides comprehensive documentation of the thermal shock testing methodology and results for all three resonator configurations. Part (a) displays the detailed temperature cycling profile used during testing, showing the temperature excursions between extreme conditions of −50 °C and +145 °C. The graph plots temperature (°C) against time, illustrating the complete thermal shock protocol where samples were maintained at each temperature extreme for 30 min before transitioning to the opposite extreme, completing a total of 10 full cycles. This rigorous testing follows industry-standard reliability assessment protocols designed to evaluate the robustness of MEMS packaging under severe thermal stress conditions. Part (b) presents a comparative bar chart showing insertion loss measurements (in dB) for all five samples of each configuration (L1, L2, and L3) both before and after the thermal shock testing. The consistent performance values between pre-test and post-test measurements conclusively demonstrate that no bonding interface failure occurred during the thermal cycling process for any of the three configurations. This thermal stability verification confirms that the Au-Au thermocompression bonding technique employed in this study provides excellent thermal resilience across all resonator width configurations, even under extreme temperature variations. These results further validate the reliability of the L3 design (100% width matching), which not only maintains superior RF performance but also exhibits robust thermal stability comparable to the other configurations, making it particularly suitable for applications in harsh environmental conditions.

### 3.5. Temperature and Humidity Test

**Figure 14:**
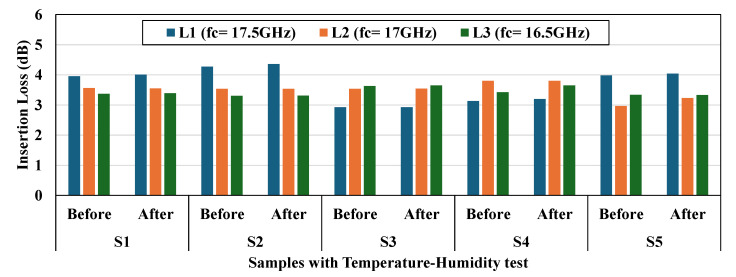
Temperature and humidity test results showing insertion loss measurements before and after environmental reliability testing according to MIL-STD-810E standards, confirming the stability of all three resonator width configurations under extreme environmental conditions.

Figure 14 presents the results of comprehensive temperature and humidity testing conducted according to rigorous military standards (MIL-STD-810E [22]) to verify the environmental reliability of all three resonator width configurations. The figure displays a detailed bar chart showing insertion loss measurements (in dB) for all five samples of each configuration (L1, L2, and L3), with paired measurements taken before and after exposure to extreme environmental conditions. Following protocols from MIL-STD-810E Method 501.4 (high temperature), MIL-STD-810E Method 502.4 (low temperature), and MIL-STD-810E Method 507.4 (humidity), the devices were subjected to extended periods of environmental stress designed to simulate real-world deployment conditions. The consistent performance values between pre-test and post-test measurements across all configurations demonstrate exceptional environmental stability, with no significant degradation in RF performance after testing. This data confirms that the Au-Au thermocompression bonding technique provides excellent resistance to temperature and humidity variations across all resonator width configurations. Most importantly, the L3 configuration (100% width matching) maintains its superior insertion loss performance even after environmental testing, demonstrating that the optimized resonator design delivers both enhanced electrical performance and environmental reliability. These results further validate the suitability of the L3 design for deployment in demanding applications where environmental stability is a critical requirement alongside optimal RF performance.

### 3.6. Performance Summary and Comparisons

Table 1 provides a performance summary in the context of other state-of-the-art wideband RF MEMS filters. It is important to note that a direct comparison of absolute metrics like insertion loss is challenging due to the wide range of operating frequencies across the reported works. Therefore, the comparison primarily serves to benchmark our filter’s fractional bandwidth, which is a more scalable figure of merit. The proposed design’s 25% fractional bandwidth represents a significant advancement, substantially exceeding the other designs, which range from 4.3% to 10%. Despite the inherent differences in filter structure (e.g., Interdigital vs. Lumped), substrate material, and operating frequency, which make a direct ‘apples-to-apples’ comparison difficult, the table provides valuable context for our work’s primary contribution. The proposed design’s 25% fractional bandwidth represents a significant advancement for wideband filter technology, offering more than double the bandwidth of the closest competing design while maintaining reasonable performance. This exceptional bandwidth-to-insertion-loss ratio positions the filter advantageously for ultra-wideband applications where wide frequency coverage is paramount.

## 4. Conclusions

This investigation establishes resonator width optimization as a critical design parameter for wideband RF MEMS filters. Through systematic evaluation of three resonator width configurations—0% (L1), 60% (L2), and 100% (L3) matching between cap and bottom wafers—this study demonstrates that complete width matching (L3) provides optimal electrical performance and mechanical reliability. The L3 configuration achieved superior RF performance with 3.34 dB insertion loss across a 4.5 GHz bandwidth (25% fractional bandwidth) and optimal loaded Q-factor (Q_L_ = 3.31). While L1 demonstrated the lowest insertion loss (3.10 dB), L3 provided enhanced electromagnetic wave coupling and minimized contact resistance at the bonding interface. Mechanical reliability testing revealed that L3 withstood forces up to 7.14 Kgf, significantly outperforming L2 (2.24 Kgf) and L1 (4.22 Kgf), representing a 218% improvement over L2. SEM analysis confirmed uniform bonding interfaces with minimal void formation (~180 nm) and tolerance to ±10 μm misalignments. Comprehensive environmental testing per MIL-STD-810E standards, including thermal cycling (−50 °C to +145 °C) and humidity exposure, validated long-term stability across all configurations. The L3 design maintained superior performance throughout environmental stress testing. Comparative analysis demonstrates exceptional 25% fractional bandwidth significantly exceeding competing designs (4.3–10%) while maintaining competitive insertion loss.

It is important to note that while the L3 configuration provides superior overall performance, its reliance on a large, perfectly matched bonding area increases its sensitivity to manufacturing tolerances, especially wafer-to-wafer alignment. This presents a classic engineering trade-off between achieving optimal performance and ensuring a wider process window for manufacturing. This fabrication approach, while specialized, enables performance levels required for demanding applications. The resulting filter is intended for use in advanced hybrid RF modules, where it can be integrated alongside other components to create a compact, high-performance system-in-package.

This research establishes that complete resonator width matching between cap and bottom wafers simultaneously optimizes Q-factor performance and mechanical bonding reliability, providing a validated framework for developing high-performance, reliable RF MEMS devices for next-generation wireless communication and sensing applications.

## Figures and Tables

**Table 1:** Performance summary of the proposed MEMS Filter and comparisons to the state of the arts of wideband RF MEMS Filters.

Parameters	This Work	[2] 2021	[5] 2021	[6] 2023	[7] 1999	[23] 2016
Substrate Material	Silicon	Aluminum	Silicon	Silicon	Silicon	Silicon
Structure	Interdigital	Lumped	Interdigital	Interdigital	Lumped	Lumped
Tunability	No	Yes	No	No	No	Yes
Center Frequency (GHz)	16	24	20.76	2.5	60	21.69
Insertion Loss (dB)	3.10	1.69	2.2	3.2	3.4	4.03
Return Loss (dB)	12.23	35	26	15	14	11.5
Fractional Bandwidth (%)	25	6.5	9.6	10	4.3	5.7

## Data Availability

Data are contained within the article.
